# Microwave Electrometry with Multi-Photon Coherence in Rydberg Atoms

**DOI:** 10.3390/s23167269

**Published:** 2023-08-19

**Authors:** Zheng Yin, Qianzhu Li, Xiaoyun Song, Zhengmao Jia, Michal Parniak, Xiao Lu, Yandong Peng

**Affiliations:** 1 Qingdao Key Laboratory of Terahertz Technology, College of Electronic and Information Engineering, Qingdao 266590, China; yzzwO1@163.com (Z.Y.); liqz_opt@163.com (Q.L.); sxygryx7301@163.com (X.S.); jiazhengmao@siom.ac.cn (Z.J.); 2 College of Electrical Engineering and Automation, Shandong University of Science and Technology, Qingdao 266590, China; luxiao98@163.com; 3 Centre for Quantum Optical Technologies, Centre of New Technologies, University of Warsaw, Banacha 2c, 02-097 Warsaw, Poland; m.parniak@cent.uw.edu.pl

**Keywords:** microwave sensing, multi-photon coherence, Rydberg atoms

## Abstract

A scheme for the measurement of a microwave (MW) electric field is proposed via multi-photon coherence in Rydberg atoms. It is based on the three-photon electromagnetically induced absorption (TPEIA) spectrum. In this process, the multi-photon produces a narrow absorption peak, which has a larger magnitude than the electromagnetically induced transparency (EIT) peak under the same conditions. The TPEIA peak is sensitive to MW fields, and can be used to measure MW electric field strength. We found that the magnitude of TPEIA peaks shows a linear relationship with the MW field strength. The simulation results show that the minimum detectable strength of the MW fields is about 1/10 of that based on an common EIT effect, and the probe sensitivity could be improved by about four times. Furthermore, the MW sensing based on three-photon coherence seems to be robust against the changes in the control field and shows a broad tunability, and the scheme may be useful for designing novel MW sensing devices.

## 1. Introduction

Atom-based metrology has been widely used in many fields, such as atomic clocks [1], and the measurement of temperature [2], frequency [3], magnetic [4] and electric fields [5], due to the unique properties of atoms and molecules. Rydberg-atom-based MW electrometry has arisen great interest [5,6]. Rydberg atoms are sensitive to electric fields, and can coherently interact with a microwave (MW) electric field. It can significantly increase the accuracy and repeatability of measurement. The main research works are based on electromagnetically induced transparency (EIT) and Aulter-Townes splitting [7,8,9,10,11], where two laser fields drive atoms to their Rydberg states, and Rydberg EIT splitting induced by a MW field is used for MW electrometry [5,12,13,14,15,16,17,18]. New achievements in MW measurement include a Rydberg-atom-based superheterodyne receiver [19], enhanced MW metrology by population repumping [20], broadband terahertz wave detection [21], the arrival angle of microwave signals [22], continuous radio frequency electric-field detection [23], extending of bandwidth sensitivity [24], auxiliary transition [25], radio-frequency phase measurements [26,27,28], etc.

Some research works concern the amplitude change in transmission spectrum. For example, MW signal strength has been proven to enhance microwave measurement in Rydberg atoms [20]. An enhanced transmission signal is obtained using self-heterodyne spectroscopy [29], and the quadratic changes in peak amplitudes demonstrates a minimum detectable RF electromagnetic field strength. We notice some interesting results in three-photon coherence [30,31,32,33], which provides a new way method for EIT-related applications, such as observation of three-photon electromagnetically induced absorption (TPEIA) [34] in atomic systems, constructive interference in the three-photon absorption [35,36], demonstration of three-photon coherence condition [37,38,39], and its extension to Rydberg atoms [40]. To the best of our knowledge, little research involves MW metrology using TPEIA in Rydberg atoms.

In this paper, we propose a scheme of measuring MW electric fields based on three-photon coherence in Rydberg atoms. A probe and control fields counter-propagate through the atomic system [41]. The ${}^{87}\mathrm{Rb}$ atoms are excited from the ground states to the Rydberg states, and the absorption spectrum of three-photon transition shows a single absorption peak around the resonant frequency. Due to microwave-induced three-photon coherence, a strong TPEIA peak appears under the three-photon resonance condition. It is interesting to find that the magnitude of the TPEIA peak changes linearly with the MW electric field strength. This scheme may be used to detect the MW electric field. The simulation results show the sensitivity could be enhanced by about four times, and the minimum detectable strength of the MW electric field could be increased by more than one order of magnitude, compared with the common EIT scheme. In the following discussion, we briefly discuss the multi-photon coherence which is consistent with the some results in Ref. [34], and pay more attention to using TPEIA to measure MW electric fields. The proposed TPEIA scheme relies on three photon coherence, and is different from the common EIT scheme related to a single-photon transition. It is also different from the MW measurement schemes in Refs. [20,29]. Our scheme shows a wide tunability and may help to design novel MW sensing devices.

## 2. Model and Methods

**Figure 1:**
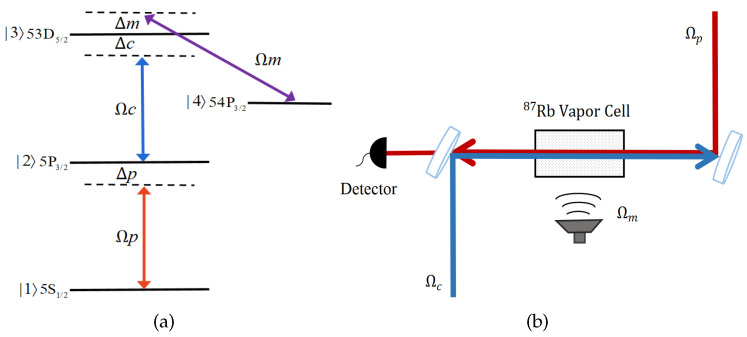
(**a**) Four-level Rydberg atom model and (**b**) schematic diagram including the atoms and coupling fields.

Figure 1a shows a four-level ladder-type atomic system [5]. The relevant atomic energy levels of ${}^{87}\mathrm{Rb}$ are $5S_{1/2}$ (|1〉), $5P_{3/2}$ (|2〉), $53D_{5/2}$ (|3〉), and $54P_{3/2}$ (|4〉). A probe laser $\Omega_{p}$ with a wavelength of $\lambda_{p}∼780$ nm and a coupling laser $\Omega_{c}$ with $\lambda_{c}∼480$ nm counter-propagate through the atoms and drive |1〉↔|2〉 and |2〉↔|3〉 transition, respectively. A MW field drives the Rydberg transition of the states |3〉 to |4〉. Figure 1b shows the schematic configuration of the coupling fields and atomic vapor cell. A similar system has been used in intracavity EIT [42], THz field measurement [43], nonlinear optical effects [44], and so on.

In the interaction picture and after the rotating wave approximation, the Hamiltonian of the system can be written as

(1) $$\begin{array}{cc} H= & -\hbar [\Delta_{p}|2\rangle \langle 2|+\left(\Delta_{p}+\Delta_{c}\right)\left|3\rangle \langle 3\right|+\left(\Delta_{p}+\Delta_{c}-\Delta_{m}\right)\left|4\rangle \langle 4\right|+\Omega_{p}\left|1\rangle \langle 2\right|\\ & +\Omega_{c}\left|2\rangle \langle 3\right|+\Omega_{m}|3\rangle \langle 4|+H.C.], \end{array}$$

where *H* is the interaction Hamiltonian, $\Omega_{p}=\mu_{12}E_{p}/\hbar$, $\Omega_{c}=\mu_{23}E_{c}/\hbar$, and $\Omega_{m}=\mu_{34}E_{m}/\hbar$ is the Rabi frequency. $\Delta_{p}=\omega_{p}-\omega_{12}$, $\Delta_{c}=\omega_{c}-\omega_{23}$, and $\Delta_{m}=\omega_{m}-\omega_{34}$ denote the detuning of the corresponding fields, respectively. $\mu_{ij}$ (*i*, *j* = 1, 2, 3, 4) is the transition dipole moment from state |i〉 to state |j〉. The dynamic evolution of the system can be described using the density-matrix method as follows [45]:

(2) $$\dot{\rho }=-\frac{i}{\hbar }\left[H,\rho \right]+L\left(\rho \right),$$

where $L\left(\rho \right)$ denotes the decoherence processes. The time evolution of density matrix elements can be written as

(3) $$\begin{array}{c} {\dot{\rho }}_{11}=\Gamma_{2}\rho_{22}-i\left(\rho_{12}-\rho_{21}\right)\Omega_{p}\;,\\ {\dot{\rho }}_{22}=-\Gamma_{2}\rho_{22}+\Gamma_{3}\rho_{33}-i\left[\left(\rho_{23}-\rho_{32}\right)\Omega_{c}+\left(\rho_{12}-\rho_{21}\right)\Omega_{p}\right]\;,\\ {\dot{\rho }}_{33}=-\Gamma_{3}\rho_{33}+\Gamma_{4}\rho_{44}-i\left[\left(\rho_{32}-\rho_{23}\right)\Omega_{c}+\left(\rho_{34}-\rho_{43}\right)\Omega_{m}\right]\;,\\ {\dot{\rho }}_{44}=-\Gamma_{4}\rho_{44}-i\left(\rho_{43}-\rho_{34}\right)\Omega_{m}\;,\\ {\dot{\rho }}_{21}=-\frac{1}{2}\Gamma_{2}\rho_{21}-i\left[\Delta_{p}\rho_{21}-\rho_{31}\Omega_{c}-\left(\rho_{11}-\rho_{22}\right)\Omega_{p}\right]\;,\\ {\dot{\rho }}_{31}=-\frac{1}{2}\Gamma_{3}\rho_{31}-i\left[\Delta_{1}\rho_{31}-\rho_{21}\Omega_{c}-\rho_{41}\Omega_{m}+\rho_{32}\Omega_{p}\right]\;,\\ {\dot{\rho }}_{41}=-\frac{1}{2}\Gamma_{4}\rho_{41}-i\left(\Delta_{2}\rho_{41}-\rho_{31}\Omega_{m}+\rho_{42}\Omega_{p}\right)\;,\\ {\dot{\rho }}_{32}=-\gamma_{32}\rho_{32}-i\left[\Delta_{c}\rho_{32}+\left(\rho_{33}-\rho_{22}\right)\Omega_{c}-\rho_{42}\Omega_{m}+\rho_{31}\Omega_{p}\right]\;,\\ {\dot{\rho }}_{42}=-\gamma_{42}\rho_{42}-i\left(\Delta_{3}\rho_{42}+\rho_{43}\Omega_{c}-\rho_{32}\Omega_{m}+\rho_{41}\Omega_{p}\right)\;,\\ {\dot{\rho }}_{43}=-\gamma_{43}\rho_{43}-i\left[-\Delta_{m}\rho_{43}+\rho_{42}\Omega_{c}+\left(\rho_{44}-\rho_{33}\right)\Omega_{m}\right], \end{array}$$

with $\rho_{ij}\;=\;{\rho^{*}}_{ji}$ and the closure relation $\sum_{j}\rho_{jj}=1$, (*i*,*j* = 1,2,3,4). Here, $\Delta_{1}=\Delta_{p}+\Delta_{c}$, $\Delta_{2}=\Delta_{p}+\Delta_{c}-\Delta_{m}$, and $\Delta_{3}=\Delta_{c}-\Delta_{m}$. $\gamma_{ij}$ is the decay from the states |i〉 to |j〉, and $\gamma_{ij}=\left(\Gamma_{i}+\Gamma_{j}\right)/2$, with $\Gamma_{i}$ being the population decay rate of state |i〉. In the weak probe field limit, we consider $\rho_{11}^{\left(0\right)}\approx 1$, $\rho_{ij}^{\left(0\right)}\approx 0$. The coherence term $\rho_{21}$ can be obtained by solving the steady-state solutions of Equation (3). With consideration of the residual Doppler effect, the frequency detuning of the control and probe fields are modified as $\delta_{c}=\Delta_{c}-k_{c}v$, $\delta_{p}=\Delta_{p}+k_{p}v$, where $k_{c}=2\pi /\lambda_{c}$, $k_{p}=2\pi /\lambda_{p}$, and the susceptibility of the Rydberg atoms is then Doppler-averaged:

(4) $$\chi =\frac{2N{\left|\mu_{12}\right|}^{2}}{\hbar \varepsilon_{0}\Omega_{p}}\frac{1}{\sqrt{\pi }u}\int_{-\infty }^{+\infty }\rho_{21}e^{-\frac{v^{2}}{u^{2}}}dv,$$

where *N* is Rydberg atom density. $\mu_{12}$ is the dipole moment of transition |1〉↔|2〉, $\varepsilon_{0}$ is the dielectric constant of vacuum, $u=\sqrt{2k_{B}T/m}$ is the most probable velocity of the atoms, $k_{B}$ is the Boltzmann constant, *T* is the temperature of system, and *m* is the mass of the atom. By solving the steady-state solution of Equation (3), we obtain the relationship between $\rho_{21}$ and $\rho_{41}$, and then the expression of the three photon coherent element $\rho_{41}$ of the density matrix in $\rho_{21}$. As a result, we obtain the three-photon coherence term in $\rho_{21}$ [34,38], and the three-photon coherence part of the atomic susceptibility is

(5) $$\chi_{\mathrm{TPC}}=\frac{2N{\left|\mu_{12}\right|}^{2}}{\hbar \varepsilon_{0}\Omega_{p}}\frac{1}{\sqrt{\pi }u}\int_{-\infty }^{+\infty }\frac{i\Omega_{m}^{2}\Omega_{c}}{C_{5}C_{6}}\frac{1}{C_{7}}\frac{i\Omega_{m}\left[C_{2}\Omega_{c}^{2}+C_{1}\left(-C_{2}C_{3}-\Omega_{m}^{2}\right)\right]+{C_{1}}^{2}\Omega_{m}^{2}\Omega_{p}^{3}\Omega_{c}}{C_{1}\Omega_{m}\Omega_{p}^{2}\left(C_{2}-C_{4}\right)C_{8}+C_{9}}dv,$$

where $C_{1}=i\Delta_{m}-G_{34}$, $C_{2}=G_{23}+i\delta_{c}$, $C_{3}=G_{24}+i\left(\delta_{c}-\Delta_{m}\right)$, $C_{4}=\Gamma_{4}/2+i\left(\delta_{c}+\delta_{p}-\Delta_{m}\right)$, $C_{5}=\Gamma_{2}/2+i\delta_{p}$, $C_{6}=\Gamma_{3}/2+i\left(\delta_{c}+\delta_{p}\right)$, $C_{7}\;=\;\left[\left(iC_{1}C_{4}C_{5}C_{6}-\Omega_{c}^{2}\right)\Omega_{m}+iC_{1}C_{5}\Omega_{m}\left(\Omega_{p}^{2}-\Omega_{m}^{2}\right)\right]$, $C_{8}\;=\;\left[C_{1}\left(C_{5}C_{6}-\Omega_{c}^{2}\right)\Omega_{m}+C_{1}C_{5}\left(\Omega_{m}-C_{5}\Omega_{c}^{2}\right)\right]$, $C_{9}\;=\;[i\Omega_{m}(C_{2}\Omega_{c}^{2}-iC_{1}C_{2}C_{3}$$+C_{1}\Omega_{m}^{2})+C_{1}\Omega_{p}^{2}]$, $G_{34}=\left(\Gamma_{3}+\Gamma_{4}\right)/2=\gamma_{34}$, and $G_{24}=\left(\Gamma_{2}+\Gamma_{4}\right)/2=\gamma_{24}$, $G_{23}=\left(\Gamma_{2}+\Gamma_{3}\right)/2\;=\;\gamma_{23}$.

## 3. Results and Discussion

**Figure 2:**
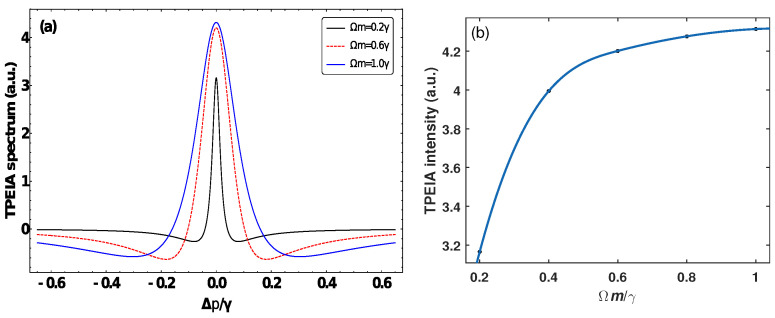
(**a**) The TPEIA spectrum and (**b**) peak intensity as a function of the MW field strength, with $\Omega_{p}=0.001\gamma$, $\Omega_{c}=3\gamma$, $\Gamma_{3}=0.2\gamma$, $\Gamma_{4}=0.01\gamma$. (γ=2π×1MHz).

We first consider the TPEIA spectrum changing with the MW field in a Doppler-free scheme, where the probe field counter-propagates with the control field (see Figure 2). The ${}^{87}\mathrm{Rb}$ atomic density is $N≃10^{7}\;cm^{-3}$ and spontaneous decay $\Gamma_{2}=6\gamma =2\pi \times 6\;\mathrm{MHz}$, $\Gamma_{3}\;=\;0.2\gamma$ and $\Gamma_{4}\;=\;0.01\gamma$ in the following discussion [5]. Furthermore, the following discussion is scaled by γ for simplicity. Figure 2a shows that the TPEIA signal has one absorption peak through the three-photon process when the lasers interact with atoms resonantly. Here, we pay attention to the variation in TPEIA with MW fields. For a weak MW field, the TPEIA peak increases with the strength of MW field. As shown in Figure 2a, the absorption peak becomes stronger with an increase in the MW field strength, and the peak linewidth becomes a little broad due to a homogeneous broadening effect. Thanks to the three-photon coherence, the population transfers from the ground state to the Rydberg state, and the peak value of absorption spectrum can be improved in the range of a weak MW field. Figure 2b shows the variations in magnitude of the TPEIA peak as a function of the MW field strength. The TPEIA peak becomes strong by increasing MW field, and the linewidth remains narrow. While the magnitude of TPEIA peak changes nonlinearly with the MW field strength, there are few changes in the TPEIA peak when the MW field strength is greater than 1γ, which may be not suitable for the linear measurement of MW electric field.

**Figure 3:**
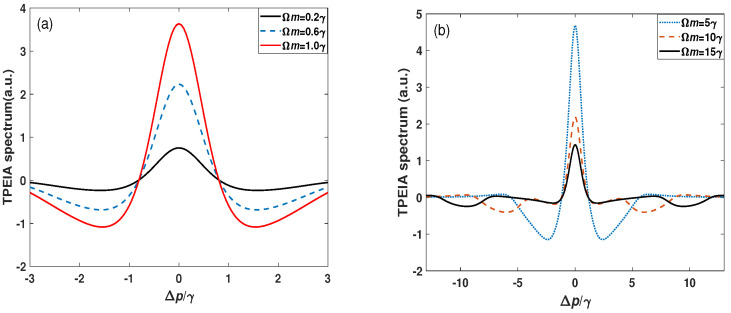
(**a**) The Doppler-averaged TPEIA spectrum and (**b**) variation in the TPEIA spectrum with MW field. The other parameters are the same as in Figure 2a.

Generally, most of the experiments are performed at room temperature, and the Doppler effect is obvious due to the atomic motion. Here a Doppler-averaged scheme is adopted, where the probe and control fields counter-propagate through the atomic vapor. The variations in the absorption spectrum are shown in Figure 3a. The TPEIA peak becomes strong with an increase in the MW field. However, when the strength of the MW field is further increased, the TPEIA signal is suppressed, and two transmission windows are far away from resonance (see Figure 3b). We pay more attention to the enhanced TPEIA peak and explore its application in precise measurement.

**Figure 4:**
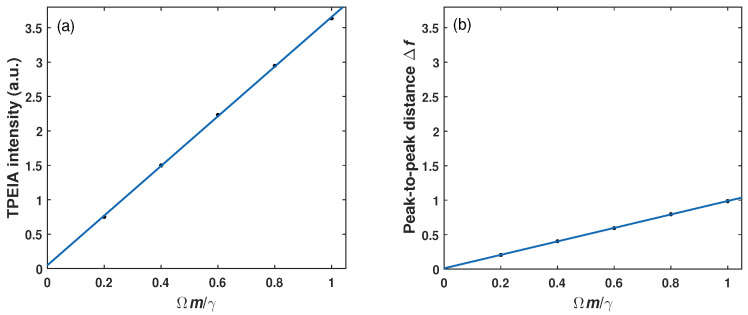
(**a**) The variations in absorption peak intensity as a function of the MW field strength on the condition of Doppler-averaged and (**b**) distance of two transmission peaks $\Delta_{f}$ versus the MW field strength based on the common EIT method. The other parameters are the same as in Figure 2a.

It is interesting to note that the magnitude of TPEIA peak varies linearly with the MW field, as shown in Figure 4a. The numerical results show that the curve slop based on three-photon coherence is about 4. Figure 4b shows the linear measurement of MW field based on the common EIT method, where the frequency splitting of EIT peaks changes linearly with the MW electric field strength. The slope of measurement curve based on EIT from the simulation is about 1. The comparison of Figure 4a,b shows that the curve slope based on TPEIA is about four times larger than that of EIT method. Different from the common EIT scheme, the MW electric field strength could be estimated from changes in the magnitude of TPEIA peaks. It is known that the larger curve slope under the same condition results in the better detection sensitivity, just as [20,31] said. This indicates that the probe sensitivity could be improved by about a factor of four, due to the three-photon coherence.

**Figure 5:**
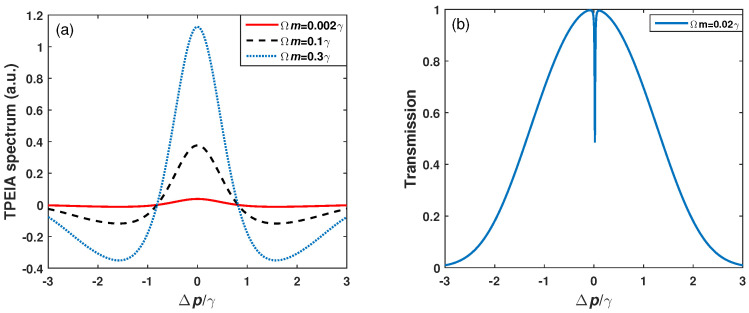
(**a**) Doppler-averaged TPEIA spectrum and (**b**) transmission spectrum of EIT. The other parameters are the same as in Figure 2a.

It is important to detect the minimum strength of the MW field for precise measurements. Figure 5a shows the minimum detectable strength $MW_{\min}$ for the three-photon resonance case. According to the Rayleigh criterion [46], the the corresponding spectrum resolution is about 0.02γ, which means the minimum detectable strength of the MW field is about 0.02γ, based on the EIT scheme, as shown in Figure 5b. Our simulation results show that the minimum detectable strength of the MW field is about 0.002γ for the TPEIA spectrum, which is about 1/10 of that based on an common EIT effect (see Figure 5a). This indicates that the minimum detectable strength could be improved by 10 times due to the three-photon coherence.

The above discussions deal with a weak MW field. In the dressed-state picture, the probe and control transition consist of a classical Rydberg EIT scheme. There is an EIT window around the resonant frequency, and it can be understood by the EIT theory [47]. The coupling fields $\Omega_{c}$ dress the states |2〉 and |3〉, and two new eigenstates appear, i.e., |+〉 and |−〉. With coupling of the probe field, two transition channels appear, |1〉→|+〉 and |1〉→|−〉. When the MW field drives the Rydberg transition |3〉→|4〉, the Rydberg-EIT is disturbed, and an enhanced absorption peak builds up, which is referred to be as TPEIA.

**Figure 6:**
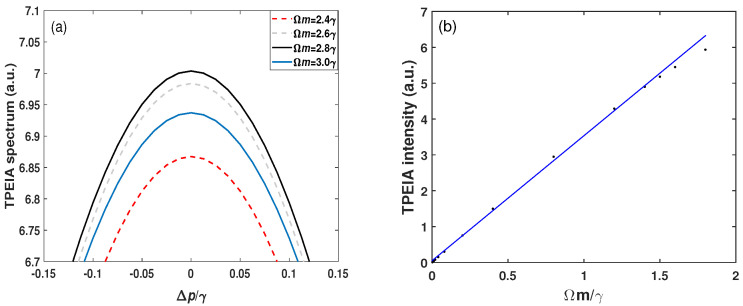
(**a**) The effect of a large MW field on the intensity of the Doppler-averaged TPEIA spectrum and (**b**) TPEIA peaks versus the MW field strength. The other parameters are the same as in Figure 2a.

Figure 6a shows the effect of a large MW field on the magnitude of the Doppler-averaged TPEIA spectrum. For example, when the MW field strength $\Omega_{m}\leq 2.8\gamma$, the TPEIA peak becomes strong with an increase in the MW field, as shown Figure 6a, while the TPEIA peak decreases with the further increase in the MW field. Thus, the TPEIA peak reaches a maximum at $\Omega_{m}=2.8\gamma$ under the given condition. This is because the two dressed states induced by the MW field are well separated, and the AT splitting of Rydberg EIT is dominant over TPEIA peak in the regime of the large MW field [40]. The constructive interference for three-photon coherence gradually weakens. When the TPEIA peak decreases with $\Omega_{m}$, the peak-to-peak distance of the two transmission peaks increases with $\Omega_{m}$, as shown in Figure 3a. This effect can be well-understood in the dressed-state picture [48]. Then, in Figure 6b, we demonstrate the dynamic range of the TPEIA scheme by measuring the magnitude of TPEIA peak as a function of the MW field strength. The TPEIA peak varies linearly over an MW field range of 0.002γ to 1.5γ from simulation. The dependence of TPEIA on the strength of the MW signal can be expressed as $\Delta h=\;2\pi \Delta \Omega_{m}\xi$, where ξ=0.58 is the enhancement coefficient of TPEIA peak under the conditions of Figure 2a, Δh and $\Delta \Omega_{m}$ are the changes in the TPEIA peak and MW Rabi frequency, respectively.

**Figure 7:**
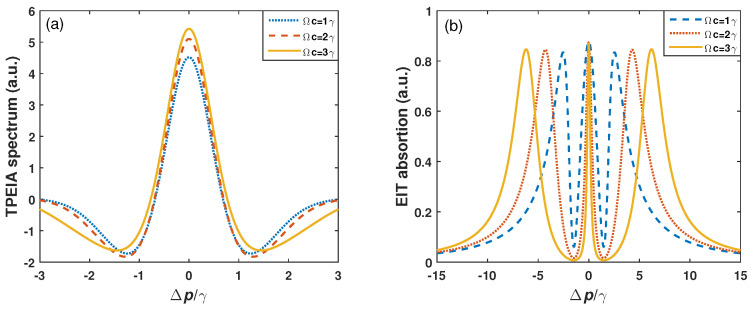
(**a**) TPEIA and (**b**) EIT spectra for different control fields, with $\Omega_{m}=1.5\gamma$. The other parameters are the same as in Figure 2a.

In addition, the numerical results show that the linewidth of the absorption spectrum is about 1.15γ. The linewidth of the TPEIA peak is broader than that of Doppler-free scheme. This is due to the residual Doppler mismatch of the probe and coupling fields. The effect of the control field on the intensity of the TPEIA spectrum is shown in Figure 7a. The magnitude of the TPEIA peak increases when the control field becomes strong. The strong control field induces good multi-photon coherence and contributes to a large TPEIA peak. Of course, if the control field is too large, the Rydberg EIT evolves into Aulter–Townes splitting, and the inter-path interference weakens, resulting in a decrease in the TPEIA peak. Figure 7b shows the effect of the control field on the linewidth of the EIT spectrum. In Figure 7b, the two EIT windows become wide when the control field builds up, and the EIT dips increase with an increase in the control field, while in Figure 7a, the linewidth of the TPEIA basically remains unchanged. This means that the TPEIA scheme shows some robustness to changes in the control field.

**Figure 8:**
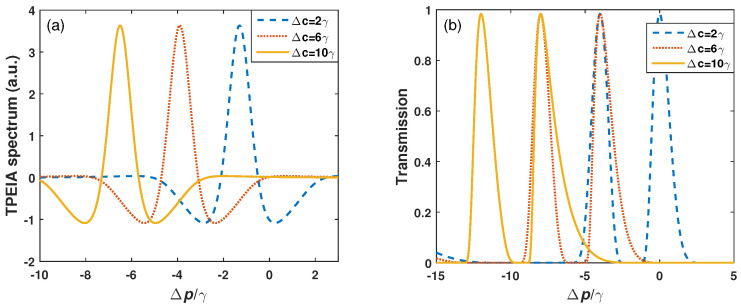
(**a**) Doppler-averaged TPEIA spectrum and (**b**) EIT spectrum for different control field detuning $\Delta_{c}$, with $\Omega_{m}=\gamma$. The other parameters are the same as in Figure 2a.

The above discussions are based on resonant interaction of the control field. Then, the detuning of the control field is considered. The effect of the control field detuning on the TPEIA and EIT spectra are shown in Figure 8. Figure 8a shows that the TPEIA peak shifts with the control field detuning $\Delta_{c}$. In this process, the TPEIA peak basically remain unchanged. This means that the TPEIA scheme has a broad detection range and some tunability, while in Figure 8b, the two transmission peaks of the EIT scheme shift with the control field detuning $\Delta_{c}$ and become asymmetric with an increase in control field detuning. For example, the linewidth of the right EIT peak increases and is greater than the left one, and the linewidth of right peak increases as the control detuning increases. In addition, when the control field detuning is 10γ, the shift of the TPEIA peak is 6.5γ from the resonance, and the shift of the EIT-AT peak is 9.3γ from the resonance. Thus, the frequency shift of the TPEIA spectrum is smaller than that of the common EIT spectrum, which means that the TPEIA scheme improves system robustness, compared to the common EIT scheme.

## 4. Conclusions

In summary, we theoretically investigated TPEIA spectrum of Rydberg atoms and proposed to use three-photon coherence to detect a weak MW electric field. Due to the multi-photon coherence, there is constructive interference in the TPEIA at the resonant frequency. It is interesting to find that the magnitude of TPEIA peaks change linearly with the MW field, which can be used to detect the MW electric field. The numerical results show the sensitivity based on TPEIA is about four times larger than that of the EIT scheme. Its minimum detectable strength is about one order of magnitude smaller than that of the EIT scheme. Moreover, the MW measurement based on TPEIA shows some robustness, a broad detection range and some tunability. The proposed scheme may help to design novel MW-sensing devices.

## Data Availability

Some or all data that support the findings of this study are available from the corresponding author upon reasonable request.
